# Silibinin suppresses bladder cancer through down-regulation of actin cytoskeleton and PI3K/Akt signaling pathways

**DOI:** 10.18632/oncotarget.20734

**Published:** 2017-09-08

**Authors:** Mitsuho Imai-Sumida, Takeshi Chiyomaru, Shahana Majid, Sharanjot Saini, Hannah Nip, Rajvir Dahiya, Yuichiro Tanaka, Soichiro Yamamura

**Affiliations:** ^1^ Department of Urology, San Francisco Veterans Affairs Medical Center and University of California, San Francisco, CA, USA; ^2^ Current address: Department of Urology, National Hospital Organization Kagoshima Medical Center, Kagoshima, Japan

**Keywords:** silibinin, bladder cancer, KRAS, PI3K, long non-coding RNA

## Abstract

Silibinin is the major active constituent of silymarin, an extract of milk thistle seeds. Silibinin has been shown to have significant anti-cancer effects in a variety of malignancies. However, the molecular mechanisms of silibinin action in bladder cancer have not been studied extensively. In the present study, we found that silibinin (10 μM) significantly suppressed proliferation, migration, invasion and induced apoptosis of T24 and UM-UC-3 human bladder cancer cells. Silibinin down-regulated the actin cytoskeleton and phosphatidylinositide 3-kinase (PI3K)/Akt signaling pathways in these cancer cell lines. These pathways were found to crosstalk through RAS cascades. We found that silibinin suppressed levels of trimethylated histone H3 lysine 4 and acetylated H3 at the *KRAS* promoter. Furthermore, silibinin targets long non-coding RNA: HOTAIR and ZFAS1, which are known to play roles as oncogenic factors in various cancers. This study shows that silibinin exerts anti-cancer effects through down-regulation of actin cytoskeleton and PI3K/Akt pathways and thus suppresses bladder cancer growth and progression.

## INTRODUCTION

Silibinin is a polyphenolic flavonolignan and is a major active component in silymarin, an extract of milk thistle (*Silybum marianum*) seeds. Silibinin has been shown to have significant anti-cancer effects in a variety of cancer types [1], including prostate [1–3], bladder [4, 5], kidney [6, 7], skin [8, 9], pancreas [10], breast [3, 11], lung [12–14] and colon [15, 16] cancers. Silibinin is also effective in inhibiting brain metastases from non-small cell lung cancer [17].

Bladder cancer is the 5th most common cancer worldwide with the lowest incidence rates in Asia and the highest rates in Western Europe and North America [18]. In the United States, bladder cancer is the 4th and 9th most common cancer in men and women respectively, comprising 7% of all cancer cases in 2014. More than 50,000 men and 16,000 women are newly diagnosed with bladder cancer in the United States in 2014 [19].

Several studies have revealed that proteins which link migratory signals to the actin cytoskeleton are upregulated in invasive and metastatic cancer cells [20]. Gain-of-function mutations in *RAS* genes are known to be the first specific genetic alterations identified in human cancers. The RAS proteins have been characterized as essential components of signaling networks controlling cellular proliferation, differentiation, or survival in association with a variety of cancers [21]. In bladder cancers, 77% of the analyzed bladder tumors expressed higher RAS levels than the surrounding normal tissues [22]. The phosphatidylinositol 3-kinase (PI3K)/Akt pathway involved in the malignant transformation of human tumors and their subsequent growth, proliferation, and metastasis. Given that PI3K/Akt signaling occupies a major place in the etiology of bladder cancers, PI3K inhibitors have potential to be used for the treatment of bladder cancers [23].

Long non-coding RNAs (lncRNAs) are a class of non-coding RNAs longer than 200 nucleotides. LncRNAs have been identified as playing critical roles in cancer. For instance, the lncRNA HOX transcript antisense RNA (HOTAIR) has been shown to facilitate tumor initiation and progression in various cancers, such as breast, gastric, colorectal, cervical, and bladder cancers [24–27]. Another lncRNA Zinc finger antisense 1 (ZFAS1) is reported to promote cancer progression in some cancers [25, 28].

This study is the first to determine the effects of a low concentration (10 μM) of silibinin on human bladder cancer cells. We identified the actin cytoskeleton and PI3K/Akt pathways as silibinin-induced down-regulated pathways, which are inter-connected through RAS. We further evaluated the effects of silibinin on histone modifications and lncRNA expression. Our results contribute to understanding the mechanisms underlying the anti-cancer effect of silibinin in bladder cancer cells.

## RESULTS

### Silibinin inhibits cell proliferation and induces apoptosis

**Figure 1:**
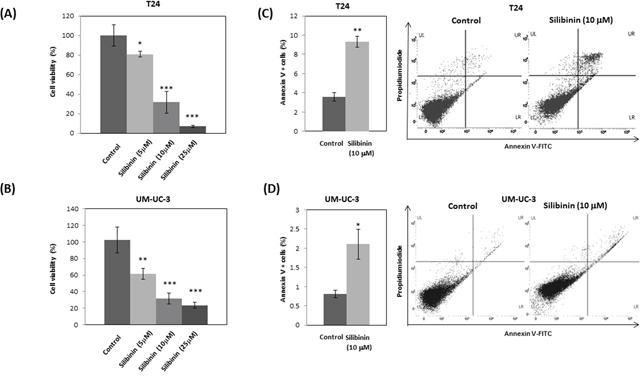
Silibinin inhibits proliferation and induces apoptosis in bladder cancer cells T24 **(A)** and UM-UC-3 **(B)** bladder cancer cells were seeded at a density of 3.78×10^4^ cells per well in 6-well plates. Silibinin at the indicated concentration (5, 10, or 25 μM) was added and cell viability was measured by cell proliferation assay at 48 hours. T24 **(C)** and UM-UC-3 **(D)** cells were incubated for 2 days and the cells were stained with AnnexinV-FITC/PI and apoptosis was analyzed by flow cytometry. One–way ANOVA followed by *Dunnett's* post hoc test (A, B). Two-tailed Student's *t*-test (C, D). Percentages in control-treated cells were defined as 100% (A, B). ^*^p < 0.05, ^**^p < 0.01, ^***^p < 0.001.

To study the effect of silibinin on the growth of bladder cancer cells, T24 and UM-UC-3 cells were treated with 5, 10 and 25 μM silibinin. Cell proliferation assay showed that silibinin inhibited T24 cell proliferation by 20% (5 μM), 69% (10 μM) and 93% (25 μM) at 48 hours (Figure 1A). Silibinin also inhibited UM-UC-3 cell proliferation by 39% (5 μM), 69% (10 μM) and 77% (25 μM) at 48 hours (Figure 1B). These results show that silibinin significantly suppresses the proliferation of these bladder cancer cell lines in a dose-dependent manner. We next studied the effects of silibinin on apoptosis in T24 and UM-UC-3 cells using Annexin-V-FITC/PI labeling. Silibinin (10 μM) increased apoptosis in both T24 and UM-UC-3 cells (Figure 1C, 1D). These results indicate that silibinin suppresses T24 and UM-UC-3 cell proliferation and increases apoptosis.

### Silibinin inhibits cell migration and invasion

**Figure 2:**
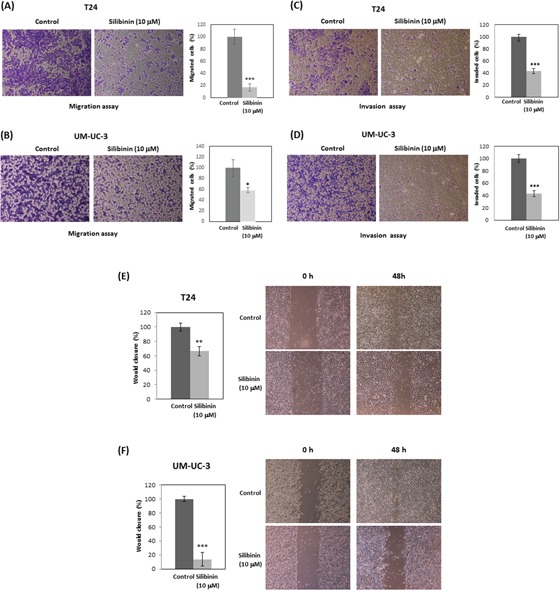
Silibinin inhibits cell migration and invasion in bladder cancer cells T24 and UM-UC-3 cells were initially incubated with silibinin (10 μM) for 48 hours for migration assays and 72 hours for invasion assays. The cells were then harvested and subjected to migration assays for 24 hours **(A, B)** or invasion assays for 48 hours **(C, D)**. T24 **(E)** and UM-UC-3 **(F)** cells were incubated with silibinin (10 μM) for 48 hours and wound healing assays were performed. Two-tailed Student's *t*-test (A-F). Percentages in control-treated cells were defined as 100% (A-F). ^*^p < 0.05, ^**^p < 0.01, ^***^p < 0.001.

T24 and UM-UC-3 cells were treated with 10 μM silibinin and subjected to Transwell migration assay without Matrigel. Silibinin reduced migration of T24 and UM-UC-3 cells to 16% and 58% of controls, respectively (Figure 2A, 2B). In addition, T24 and UM-UC-3 cells were treated with 10 μM silibinin and subjected to Transwell invasion assay with Matrigel. Silibinin reduced invasion of T24 and UM-UC-3 cells to 43% and 42% of controls, respectively (Figure 2C, 2D). Wound-healing assay also showed that silibinin (10 μM) reduced the migration of T24 and UM-UC-3 cells to 66% and 13% of controls at 48 hours, respectively (Figure 2E, 2F). Our results show that silibinin (10 μM) effectively inhibits cell migration and invasion in bladder cancer cells.

### Silibinin down-regulates RAS, PI3K/Akt and actin cytoskeleton pathways

**Figure 3:**
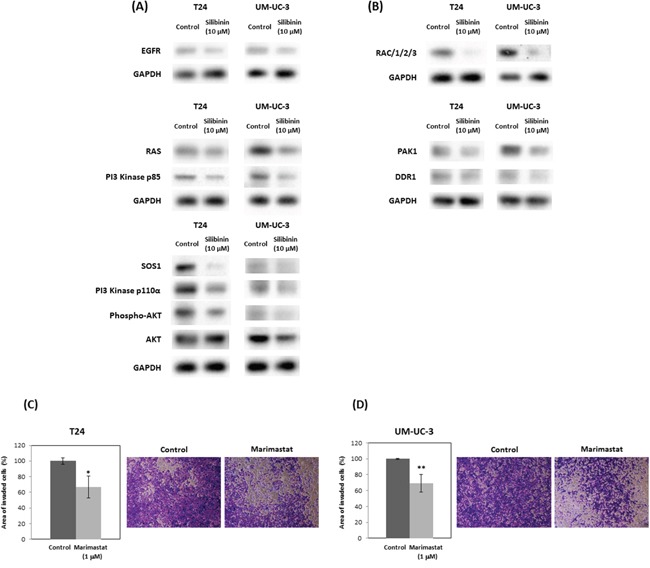
Silibinin down-regulates expression of genes in PI3K/Akt signaling and actin cytoskeleton pathways Expression of genes in PI3K/Akt signaling pathway **(A)** and actin cytoskeleton pathways **(B)** were assayed by Western blot following 10 μM silibinin treatment for 72 hours. T24 **(C)** and UM-UC-3 **(D)** cells were incubated with marimastat (1 μM) for 48 hours and the cells were harvested and subjected to Transwell invasion assay with Matrigel. EGFR: Epidermal Growth Factor Receptor; SOS1: Son of Sevenless Homolog 1, Rac: Ras-Related C3 Botulinum Toxin Substrate, PAK1: P21 Protein (Cdc42/Rac)-Activated Kinase 1, DDR1 (RTK): Receptor tyrosine kinases. Two-tailed Student's *t*-test (C, D). Percentages in control-treated cells were defined as 100% (C, D). ^*^p < 0.05, ^**^p < 0.01.

Western blot analysis showed that silibinin suppressed RAS and related-proteins such as epidermal growth factor receptor (EGFR) and Son of sevenless homolog 1 (SOS1) (Figure 3A). In response to EGF, SOS1 interacts with activated EGFR, through the adaptor protein, leading to the activation of RAS through the juxtaposition of SOS1 and RAS at the membrane [29, 30]. Western blot data also showed that silibinin decreased the expression of PI3K subunits such as p110 α and p85 (Figure 3A), which regulate Akt activation and Rac signals leading to actin cytoskeleton pathways. Consistently, silibinin also reduced Akt phosphorylation at Ser-473, Rac levels and the downstream signaling element of Rac (Figure 3A). Silibinin also down-regulated actin cytoskeleton pathway-related proteins such as Rac, PAK1, and discoid in domain receptor 1 (DDR1) (Figure 3B). Related to the actin-cytoskeleton, MMP activity is known to participate in actin cytoskeletal reorganization in various cell types [31]. In support of this, we confirmed that the MMP inhibitor marimastat, reduced invasion of T24 and UM-UC-3 cells to 66% and 68% of controls, respectively (Figure 3C, 3D).

### KRAS mediates silibinin functions

**Figure 4:**
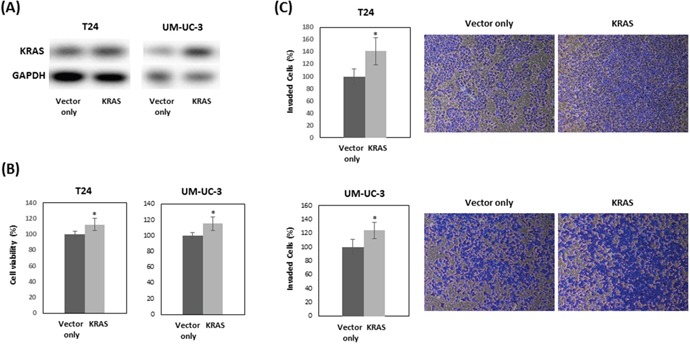
KRAS restores cell proliferation which is suppressed by silibinin T24 and UM-UC-3 cells were treated with 10 μM silibinin after transfection with vector only or vector expressing KRAS for 48 hours. KRAS expression was analyzed by Western blot **(A)**. Cell viability **(B)** and cell invasion **(C)** were evaluated at 48 hours after treatment with 10 μM silibinin. Two-tailed Student's *t*-test (B, C). Percentages in control-treated cells were defined as 100% (B, C). ^*^ *p* < 0.05.

To examine if the effects of silibinin on cell proliferation and invasion are mediated by KRAS, T24 and UM-UC-3 cells were transfected with human KRAS and treated with 10 μM silibinin. Western blot analysis confirmed that KRAS transfection increased KRAS expression in silibinin-treated cells (Figure 4A). Cell proliferation assays showed that KRAS restored cell viability (Figure 4B), which was suppressed by silibinin (Figure 1A, 1B). Invasion assays showed that KRAS promoted invasion of silibinin-treated T24 and UN-UC-3 cells (Figure 4C), which was attenuated by silibinin (Figure 2C, 2D).

### Silibinin down-regulates *KRAS* mRNA expression and suppresses histone H3 lysine 4 (H3K4) trimethylation (H3K4me3) and H3 acetylation (AcH3)

**Figure 5:**
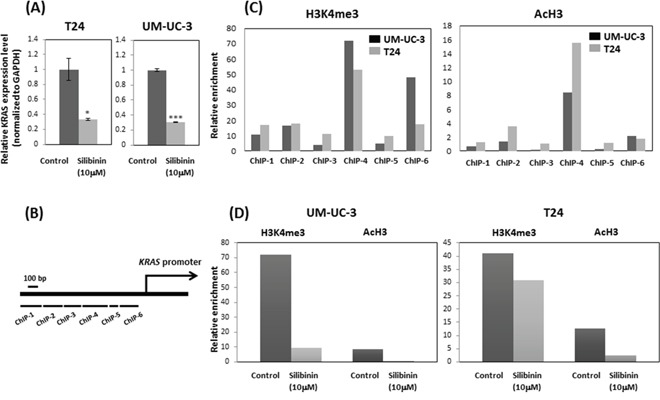
Silibinin suppressed histone H3 lysine 4 (H3K4) trimethylation (H3K4me3) and H3 acetylation (AcH3) and down-regulated KRAS expression **(A)** Relative *KRAS* mRNA expression after 10 μM silibinin treatment for 48 hours. **(B)** Schematic representation of the promoter region of the human *KRAS* gene and primer set location. **(C)** ChIP assays were performed to detect Ras levels at multiple primer sets (ChIP 1-ChIP 6). **(D)** The relative enrichment of H3K4me3 and AcH3 at ras primer-4 (ChIP-4) decreased after silibinin treatment for 48 hours. Two-tailed Student's *t*-test (A). Expression levels in control-treated cells were defined as 1 (A). ^*^p < 0.05, ^***^p < 0.001.

Expression levels of *KRAS* mRNA were significantly down regulated in T24 and UM-UC-3 cells after treatment with silibinin (10 μM) compared with vehicle-treated control cells (Figure 5A). To determine whether *KRAS* mRNA down-regulation by silibinin is linked to histone H3K4me3 at the *KRAS* gene promoter, we performed chromatin immunoprecipitation (ChIP)-quantitative real-time PCR (qPCR) assay using T24 and UM-UC-3 cells. ChIP-qPCR assays revealed that histone H3K4me3 was recruited to the ChIP-4 region of the *KRAS* gene promoter (Figure 5B, 5C). We further demonstrated that recruitment of histone H3K4me3 to the *KRAS* promoter ChIP-4 region was attenuated by silibinin treatment (Figure 5D). Similar results were obtained with AcH3 (Figure 5C, 5D). Given that methylation at lysine 4 of H3K4 and H3 hyperacetylation are associated with active transcription [19], these results indicate that silibinin suppressed the recruitments of histone H3K4me3 and AcH3 to the *KRAS* promoter, which results in repression of *KRAS* gene transcription.

### Silibinin regulates expression of long non-coding RNA

**Figure 6:**
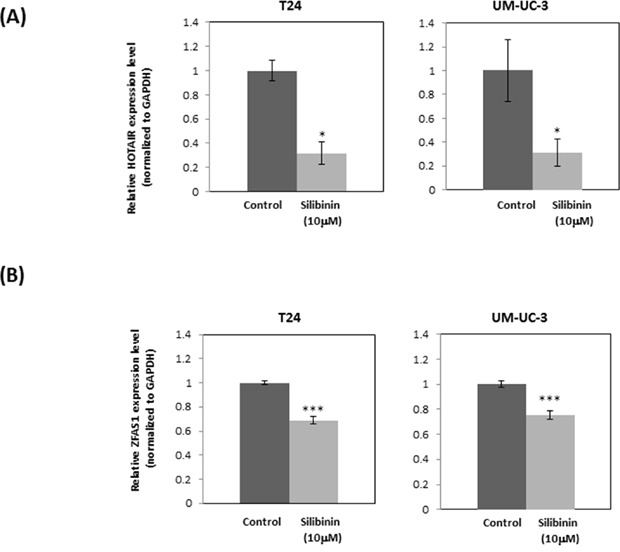
Silibinin down-regulates expression of oncogenic lncRNAs: HOTAIR and ZFAS1 Expression of long non-coding RNA HOTAIR **(A)** and ZFAS1 **(B)** after treatment of T24 and UM-UC-3 cells with silibinin (10 μM) for 4 hours as determined by real-time PCR (mean ± SD). *GAPDH* was used as an internal control. Data were obtained from three independent experiments performed in triplicate. Two-tailed Student's *t*-test (A, B). Expression levels in control-treated cells were defined as 1 (A, B). ^*^p < 0.05, ^***^p < 0.001.

To study the effect of silibinin on lncRNAs which have been shown to regulate bladder cancer, we treated bladder cancer cell lines with silibinin and assayed levels of tumor-related lncRNAs. Silibinin was found to significantly decrease the expression levels of oncogenic HOTAIR and ZFAS1 (Figure 6A, 6B). Silibinin (10 μM) had no significant effects on the levels of other lncRNAs such as metastasis-associated lung adenocarcinoma transcript 1 (MALAT1), maternally expressed 3 (MEG3), and growth arrest specific 5 (GAS5) (Supplementary Figure 1). These data indicate that silibinin exerts its effects via modulating oncogenic lncRNAs expression. As described above, silibinin may exert its anti-cancer effects by down-regulating RAS-driven actin cytoskeleton and PI3K/Akt pathways. PI3K is considered one of the main effector pathways of RAS. Thus, we investigated the relationship between the PI3K pathway and lncRNAs using the PI3K inhibitor, wortmannin. Wortmannin significantly decreased the expression level of HOTAIR in UM-UC-3 cells and showed a similar tendency in T24 cells (Supplementary Figure 2A). However, wortmannin had no effect on ZFAS1 expression in both UM-UC-3 and T24 cells (Supplementary Figure 2B).

## DISCUSSION

We found that silibinin (10 μM), at lower concentrations than previously reported (50–100 μM) [4, 5, 32], inhibits cell growth, cell migration, cell invasion, and induces apoptosis in human bladder cancer cell lines. Silibinin down-regulates expression of genes in the actin cytoskeleton and PI3K/Akt pathways. We also show that silibinin suppressed RAS expression, which is regulated by histone modification (H3K4me3 and AcH3). Moreover, silibinin suppressed the expression of lncRNA HOTAIR and ZFAS1, which are reported to promote several types of cancers.

The cytoskeleton maintains the shape and internal organization of cells, and also functions in cell division and migration. Cell migration is initiated by an actin-dependent protrusion of the cell's leading edge, which is composed of structures called lamellipodia and filopodia. Therefore, actin and the cytoskeleton are fundamental for cell migration, a critical step in cancer invasion and metastasis. Rac-PAK1 signaling, down-regulated by silibinin (Figure 3B), is known to be critical for the cytoskeleton [33]. In addition, there is evidence for the importance of the RAS-driven Rac–PAK1 effector signaling pathway in cancer development and growth [33]. DDR1 which was reduced by silibinin (Figure 3B) triggers epithelial cell differentiation by promoting cell adhesion [34]. Given the literature and our finding that silibinin significantly down-regulates actin cytoskeleton-related Ras-Rac-PAK1 pathways (Figure 3A, 3B), the inhibitory effects of silibinin on cell migration *in vitro* might be explained by reduced cytoskeleton organization and/or dynamics. This is supported by studies on the relationship between MMPs and actin cytoskeletal reorganization [31] and our results from invasion assay using the MMP inhibitor marimastat.

PI3K signaling promotes cellular transformation and cancer development, and controls the actin-cytoskeleton pathway [35] through regulating RAS [36]. PI3K is one of the main effector pathways of RAS, regulating cell growth, cell survival, and cytoskeleton reorganization [37]. Rac is also a key downstream effector of PI3K [38]. Thus, our Western blotting data showing that silibinin down-regulates both RAS and PI3K/Akt is consistent with these findings. The actin cytoskeleton pathway was also reported to crosstalk through PI3K in an AKT-independent manner [39]. In the presence of silibinin, KRAS overexpression restored cell proliferation and invasion which were suppressed by silibinin in bladder cancer cells (Figure 4B, 4C). These results support the idea that suppressed cell proliferation and invasion by silibinin are mediated by KRAS. Our ChIP data shows that histone modifications regulate *KRAS* gene expression. Histone H3K4me3 and AcH3 were enriched at the *KRAS* promoter, and silibinin suppressed the enrichment of histone H3K4me3 and AcH3 at the *KRAS* promoter.

Recently, accumulating studies have demonstrated that lncRNAs are associated with the development of various types of cancers [40]. HOTAIR has been found to be highly expressed in several cancers and related to the recurrence of bladder cancer [27]. KRAS upregulates HOTAIR [41] and is regulated by the PI3K pathway [42]. This is strongly supported by our results showing that PI3K inhibitor wortmannin downregulated HOTAIR expression in human bladder cancer cells (Supplementary Figure 2A). These findings are consistent with our findings showing that silibinin down-regulated the PI3K pathway (Figure 3A), KRAS (Figure 5A) and HOTAIR (Figure 6A).

In summary, this study shows that 10 μM silibinin induces apoptosis and inhibits cell growth, migration and invasion in human bladder cancer cells. Silibinin significantly down-regulates the RAS-driven actin cytoskeleton and PI3K/Akt pathways. Our data shows that silibinin down-regulates KRAS by suppressing histone H3K4 and AcH3 enrichment at the *KRAS* promoter. Furthermore, silibinin may exert its anti-cancer effects by suppressing oncogenic lncRNAs such as HOTAIR and ZFAS1. Hence, these pathways down-regulated by silibinin cooperatively exert tumor suppressing effects in bladder cancer cells. Currently, only a minority of patients with advanced bladder cancer are cured following treatment with standard therapies. This study may thus be important for development of silibinin as a drug for bladder cancer treatment.

## MATERIALS AND METHODS

### Regents

Silibinin, marimastat, and wortmannin were purchased from Sigma–Aldrich (St. Louis, MO, USA). RIPA buffer was purchased from Cell Signaling Technology (Boston, MA, USA), protease inhibitor and phosphatase inhibitor were from Roche (Basel, Switzerland). BCA qualification system was purchased from Pierce (Rockford, IL, USA). The following antibodies were purchased from Cell Signaling Technology (Danvers, MA): PI3 Kinase p110α (4249), PI3 Kinase p85 (4257), Akt (4691), phospho-Akt (Ser473) (Ser473) (4060), Ras (3339), SOS1 (12409), PAK1 (2602), Rac 1/2/3 (2465), DDR1 (RTK6) (5583) and GAPDH (97166).

### Cell culture

Human bladder carcinoma cell lines, T24 and UM-UC-3 were purchased from The American Type Culture Collection (Manassas, VA). T24 cells were cultured in McCoy's 5A medium and UM-UC-3 cells were cultured in Eagle's Minimum Essential medium. Both were supplemented with 10% fetal bovine serum (FBS) and 1% penicillin-streptomycin in a humidified atmosphere of 5% CO_2_ and 95% air at 37°C. Silibinin, marimastat, and wortmannin were dissolved in dimethyl sulfoxide (DMSO). The final concentration of DMSO in the culture did not exceed 0.1% (v/v).

### Transfection

KRAS expression vector was purchased from Origene (Rockville, MD). Transfection was performed using X-tremeGENE HP DNA Transfection Reagent (Roche) according to the manufacturer's protocols.

### Cell proliferation assay

Cells (T24 and UM-UC-3) were seeded at a density of 3.78 ×10^4^ cells per well in 6-well plates. After 48 hours treatment with silibinin, cell proliferation/viability was assessed by counting cell numbers with a microscope or by using CellTiter 96 Aqueous One Solution Cell Proliferation Assay (Promega, Madison, WI), a colorimetric assay which measures the activity of reductase enzymes. At the indicated times, CellTiter 96 Aqueous One reagent was added to each well according to the manufacturer's instructions. Cell viability was determined by measuring the absorbance at 490 nm using a kinetic microplate reader (Spectra MAX 190; Molecular Devices Co., Sunnyvale, CA). Data are the mean ± standard deviation (SD) of three independent experiments.

### Apoptosis analysis

Cells (T24 and UM-UC-3) were seeded at a density of 1×10^6^ cells per well in 6-well plates. After 48 hours treatment with silibinin, apoptotic cells were measured using flow cytometry (Cell Lab Quanta SC, Beckman Coulter, Brea, CA) with Annexin-V-FITC/PI labeling (BD Pharmingen, San Diego, CA). Measurements were repeated independently three times.

### Transwell invasion/migration assay

T24 and UM-UC-3 cells were grown in Dulbecco's Modified Eagle's Medium (DMEM) containing 10% FBS. For invasion assay, culture inserts of 8-μm pore size (Transwell; Corning Costar, Corning, NY) were coated with Matrigel (BD Biosciences, San Jose; 100 μg per well). For migration assay, no Matrigel was used. Inserts were placed into the wells of 24-well culture plates. In the lower chamber, 500 μl of DMEM containing 10% FBS was added. The cells which were pretreated with silibinin (10 μM) for 48 hours for migration assay and for 72 hours for invasion assay were seeded to the upper chamber. After incubation for 24 hours for migration assay and 48 hours for invasion assay at 37°C with 5% CO_2_, the cells that had migrated through the pores were fixed with 10% formaldehyde and stained with 0.05% Crystal Violet. Crystal Violet was solubilized with methanol and absorbance (540 nm) of the solution was measured by a kinetic microplate reader (Spectra MAX 190; Molecular Devices Co., Sunnyvale, CA). Data are the mean ± S.D. of three independent experiments.

### Wound-healing assay

T24 (3×10^5^) and UM-UC-3 (7×10^5^) cells were seeded in 6-well plates and treated with silibinin (10 μM) for 48 hours. A wound was formed by scraping the cells with a 200-μl pipette tip and was washed twice with medium. Cells were observed and photographed with a microscope at various times after scraping.

### Western blot analysis

Protein extracts were resolved by sodium dodecyl-sulfate polyacrylamide gel electrophoresis (SDS-PAGE) and transferred to polyvinylidene fluoride membranes (Hybond-P; GE Healthcare, Piscataway, NJ), followed by incubation with the indicated primary and secondary antibodies conjugated to horseradish peroxidase (GE Healthcare). Signals were detected using the ECL detection system (Amersham ECL plus Western Blotting detection system, Fairfield, CT).

### ChIP assay

ChIP analysis was performed using the EZ-Magna ChIP kit (Millipore) according to the manufacturer's directions. In short, cells were incubated with 1% formaldehyde for 10 min at room temperature and quenched unreacted formaldehyde with 125 mM glycine for 5 min. The cells were washed twice with phosphate buffered saline and subjected to chromatin preparation according to the kit procedure. ChIP was performed using antibodies against Tri-methyl-histone H3K4 (9751, Cell Signaling), Tri-methyl-histone H3K27 (9733, Cell Signaling), acetyl-histoneH3 upstate (06-599, Millipore), RNA polymerase II antibody (05-623B, Millipore), and an antibody against mouse IgG (12-371B, Millipore) was used as a negative control. Precipitated genomic DNA was amplified by real-time PCR. PCR was performed using STBR Green PCR master mix according to the manufacturer's suggestions (Qiagen, Valencia, CA, USA). The primer list is shown in Supplementary Table 1.

### Total RNA and protein extraction

Total RNA was extracted from cultured bladder cell lines using miRNeasy Mini Kit (Qiagen) according to the manufacturer's instructions. Cells were lysed with RIPA buffer (Thermo Scientific, Pierce) containing protease and phosphatase inhibitor cocktail (Thermo Scientific).

### Reverse transcription and real-time PCR

cDNA was synthesized using an iScript Synthesis Kit (Bio-Rad, Hercules, CA, USA). QPCR analysis was performed in triplicate with an Applied Biosystems Prism 7500 fast Sequence Detection System using Taqman universal PCR master mix fast according to the manufacturer's protocol (Applied Biosystems Inc., Foster City, CA, USA). The Taqman probes and primers for HOTAIR, ZFAS1, MALAT1, MEG3, and GAS5 were purchased from Applied Biosystems. Human GAPDH were used as an internal control. PCR primers used in QPCR assays are listed in Supplementary Table 1. Levels of RNA expression were determined by using the 7500 Fast System SDS software version 1.3.1 (Applied Biosystems).

### Statistical analysis

Data are shown as mean values ± standard deviation (SD) or standard error of the mean (SEM). The Student's *t*-test was used to compare the two different groups. *P* values of less than 0.05 were regarded as statistically significant.

## SUPPLEMENTARY MATERIALS FIGURES AND TABLES



## Abbereviations

AcH3: H3 acetylation
DDR1: discoid in domain receptor 1
EGFR: epidermal growth factor receptor
GAS5: growth arrest specific 5
H3K4me3: histone H3 lysine trimethylation
HOTAIR: HOX transcript antisense RNA
MALAT1: metastasis-associated lung adenocarcinoma transcript 1
MEG3: maternally expressed 3
PAK1: P21 Protein (Cdc42/Rac)-Activated Kinase 1
PI3K/Akt: phosphatidylinositol 3-kinase
Rac: Ras-Related C3 Botulinum Toxin Substrate
SOS1: Son of sevenless homolog 1
ZFAS1: Zinc finger antisense 1
