# Supplementary material for: Silibinin suppresses bladder cancer through down-regulation of actin cytoskeleton and PI3K/Akt signaling pathways
Source: Oncotarget. 2017 Sep 8;8(54):92032–42. doi: 10.18632/oncotarget.20734 (PMC5696161; doi:10.18632/oncotarget.20734)
Supplement: Supplementary file 1 [file oncotarget-08-92032-s001.pdf]

Silibinin suppresses bladder cancer through down-regulation of actin cytoskeleton and PI3K/Akt signaling pathways

SUPPLYMENTARY MATERIALS

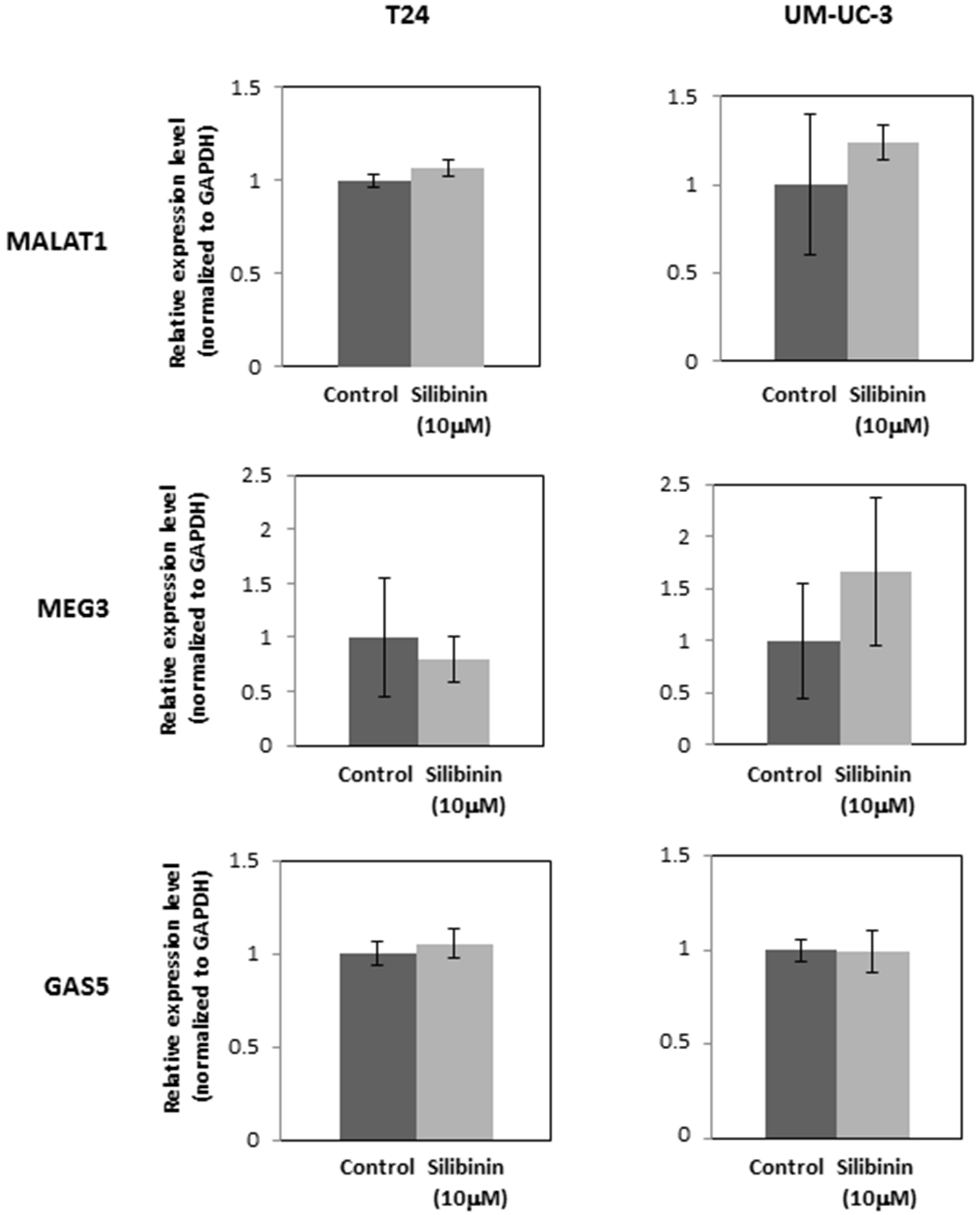

Supplymentary Figure 1: Effect of silibinin on lncRNAs: MALAT1, MEG3, and GAS5 in T24 and UM-UC-3 cells. Expression of long non-coding RNA, MALAT1, MEG3, and GAS5after treatment with silibinin (10 μM) for 4 hours was determined by real-time PCR (mean ± SD). GAPDH was used an internal control. Expression levels in control-treated cells were defined as 1.

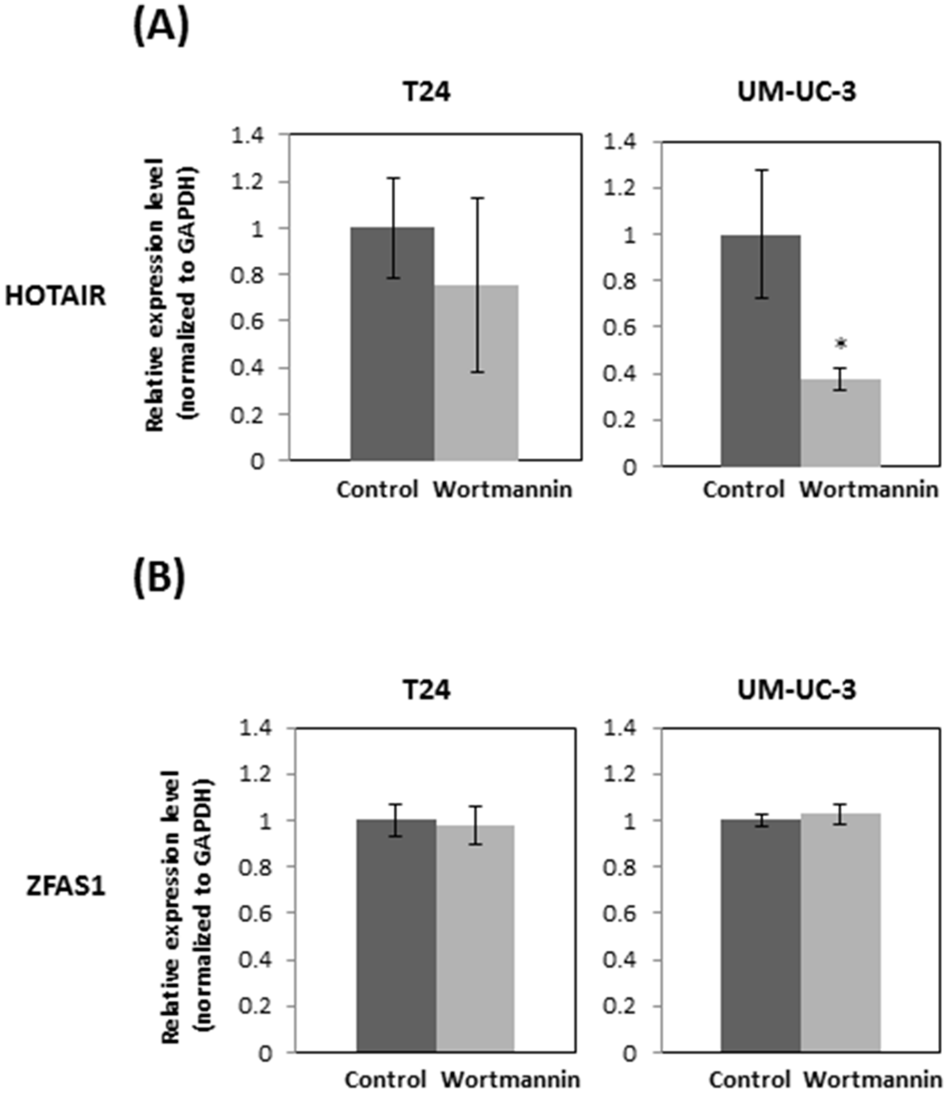

Supplymentary Figure 2: Effect of wortmannin on lncRNAs: HOTAIR and ZFAS1 in T24 and UM-UC-3 cells. Expression of long non-coding RNA, HOTAIR **(A)** and ZFAS1 **(B)**, after treatment with wortmannin (1 μM) for 24 hours was determined by real-time PCR (mean ± SD). GAPDH was used an internal control. Expression levels in control-treated cells were defined as 1.

Supplymentary Table 1: Primer oligonucleotide sequences for ChIP assays

	Primer sequence (5'-3')	Product size (bp)
ChIP-1-F	TTGAATGACTCTCCTCAGGTCT	214
ChIP-1-R	GGCCCAAACAAACACGACAG	
ChIP-2-F	CTGTCGTGTTTGTTTGGGCC	196
ChIP-2-R	TGGCGCGCATCCATTTACTA	
ChIP-3-F	TAGTAAATGGATGCGCGCCA	174
ChIP-3-R	GCAGCCGCCAATTCTGAC	
ChIP-4-F	GTCAGAATTGGCGGCTGC	254
ChIP-4-R	GTGTGGGAAGAGGGAAGAG	
ChIP-5-F	CTCTTCCCTCTTCCCACAC	88
ChIP-5-R	AGAGCTATCGATGCGTTCCG	
ChIP-6-F	CGGAACGCATCGATAGCTCT	188
ChIP-6-R	CTCGCTCCCAGTCCGAAATG	
